# The misuse of Cyproheptadine: a non-communicable disease risk behaviour in Kinshasa population, Democratic Republic of Congo

**DOI:** 10.1186/s13011-016-0051-8

**Published:** 2016-02-09

**Authors:** Aimée M. Lulebo, Carine D. Bavuidibo, Eric M. Mafuta, Josaphat D. Ndelo, Lievin’s Corneille M. Mputu, Dalton M. Kabundji, Paulin B. Mutombo

**Affiliations:** Kinshasa School of Public Health, School of Medicine, University of Kinshasa, Kinshasa, Democratic Republic of Congo; DKT, Matadi, Kongo Central Democratic Republic of Congo; School of Pharmacy, University of Kinshasa, Kinshasa, Democratic Republic of Congo; Department of Family Medicine, Faculty of Health Sciences, University of the Witwatersrand, Johannesburg, South Africa; Department of Epidemiology and Biostatistics, Kinshasa School of Public Health, School of Medicine, University of Kinshasa, Po Box 11850, Kinshasa, Democratic Republic of the Congo

**Keywords:** Cyproheptadine, Weight gain, Non-communicable disease, Obesity, Risk behaviour

## Abstract

**Background:**

Obesity is one of the main risk factors of non-communicable diseases (NCDs) worldwide, especially in sub-Saharan Africa. The use of Cyproheptadine increases body weight and the risk of becoming obese. The aim of this study is to determine the prevalence of Cyproheptadine misuse in the Kinshasa population and to describe its characteristics.

**Methods:**

A cross-sectional study was conducted in two town sectors of Kinshasa, Democratic Republic of Congo (DRC), over a 4 month period (May 2011 to August 2011). Data from 499 participants, aged between 13 and 55 years were collected and analyzed. Mean and standard deviation were used for quantitative variables and frequency and percentage for categorical variables. In order to determine the relationship between socio-demographic status and Cyproheptadine use the Chi-square test was conducted. Student’s *t*-test was used to compare means age of Cyproheptadine users and non-users. Logistic regression was used to determine predictors of Cyproheptadine use. A p-value of <0.05 was considered statistically significant.

**Results:**

Overall, 499 participants were enrolled (352 females, 147 males, mean age ± standard deviation 24.9 ± 9.7 years) in the study. The majority of the study participants (72.9 %) had used Cyproheptadine as an appetite stimulant. Females were 11 times more likely to use Cryproheptadine (OR = 11.9; 95 % CI: 7.1–20.1) than males. People aged between 36 and 55 were three times less likely to use Cryproheptadine (OR = 0.3; 95 % CI: 0.2–0.8) compared to teenagers. More than half of the participants (69.0 %) declared to take daily Cyproheptadine. Half of the study participants (50.0 %) used Cyproheptadine for more than a year and also declared to combine it with Dexamethasone (87.6 %).

**Conclusion:**

This study shows that the Kinshasa population is significantly misusing Cyproheptadine and is highly exposed to its risk, including obesity.

## Background

The World Health Organization (WHO) estimates that, within the next few years, non-communicable diseases (NCDs) will become the principal global causes of morbidity and mortality worldwide [1]. Obesity is among the main risk factors of NCDs such as cardiovascular diseases (CVDs), including arterial hypertension, Type-2 Diabetes Mellitus, and metabolic syndrome; chronic kidney disease, osteoarthritis and cancers [2–4]. Its prevalence worldwide has doubled during the past 30 years, females being more obese than males [5].

In sub-Saharan African (SSA) countries, obesity has also become an important public health problem. This is mainly due to the changing lifestyle of the population in relation to urbanization [6, 7]. The study by Dalal et al. on NCDs in SSA reported a range of obesity prevalence between 0.4 and 41 % [8]. This wide range of proportions can be explained by the study methods used as well as the population studied. During 2007, the prevalence of obesity in the DRC was estimated to be 12.2 % for males and 13.3 % for females [9]. Studies carried out in the DRC showed that obesity was a significant predictor of mortality among hypertensive patients and a significant predictor of chronic kidney diseases [10, 11]. This shows that obesity is a major public health issue in the country. Therefore, knowledge of each risk factor leading to obesity is important in managing modifiable NCDs risk factors.

Several factors have been reported as risk factors of obesity. These include genetic predisposition, diet, physical activity, gender, cultural norms, and physical environment [3]. The most challenging is that several African societies do not recognize obesity as a health problem, but as a display of social well being and beauty [12–14]. For example, a study conducted in a multi-ethnic Caribbean population showed that overweight African women were more likely to be satisfied with their body image than non-African overweight women [15]. It is reported too that overweight and obese African women do not consider themselves as such, thereby reducing the likelihood of them attempting to lose weight [16].

In Kinshasa, a new phenomenon named/known as the “C4 phenomenon”, which means that the use of Cyproheptadine for weight gain and for obtaining a “roundness physical appearance,” has been promoted among the general population. C4 is a commercial name for Cyproheptadine, a first-generation antihistamine with anti-serotonergic activity indicated for treatment of rhinitis, conjunctivitis and urticaria [17] but which has appetite stimulation as a side effect thereby increasing food intake and inducing weight gain. It is by using this side effect that Cyproheptadine is prescribed in the treatment of anorexia, cachexia, and severe malnutrition. The effect of Cyproheptadine on weight gain has been confirmed in many studies. For example, a randomized controlled trial by Couluris et al. of the effect of Cyproheptadine hydrochloride on weight in children with cancer/treatment-related cachexia found a mean weight gain of 2.6 Kg after 4 weeks of treatment with Cyproheptadine among patients with cachexia [18]. Interestingly, several others randomized controlled trials strengthened the effect of Cyproheptadine on weight gain in both children and adults’ populations. These studies reported a high average weight gain in the group of patients receiving Cyproheptadine than in the placebo group [19, 20]. However in Kinshasa, this molecule is abusively promoted, labeled and provided as an appetite stimulating drug and is sold to people who desire to gain weight, even without medical prescription, in order to have “roundness physical appearance”. This inappropriate use of Cyproheptadine can lead unconsciously to obesity while users aim for roundness. Currently no study has been carried out for describing this non-communicable disease risk behaviour in the Kinshasa population. This study aims to determine the prevalence of the misuse of Cyproheptadine in the Kinshasa population and to describe its characteristics.

## Methods

### Study design and sample

Between May and August 2011 a cross-sectional study was carried out in two town sectors of Kinshasa. These town sectors were selected purposively because of their location in the central part of Kinshasa and their population behaviour to the up-to-date fashion. Thereafter, a multi-stage sampling was used for identifying study participants. The first stage involved selection of three neighborhoods in each town sector using a simple random sampling technique. In the second stage, in each selected neighborhood three streets were selected using also a simple random sampling technique. In the streets by systematic random sampling the household was selected and there all members of household aged between 13 and 55 years were enrolled. A total of 499 participants were surveyed. The study protocol was approved by the Internal Review Board of the University Of Kinshasa Faculty Of Pharmacy (Protocol approval number 05/2011). A written informed consent according to the Helsinki declaration II was obtained from all the study participants. For participants younger than 16 years of age, signed informed consent of parents were additionally obtained. Confidentiality was maintained by not using participants’ names in the questionnaire. A face-to-face interview using a structured questionnaire was used for data collection.

### Study variables

The content of the first part of the questionnaire included socio-demographic variables (gender, age, education level, and marital status); followed in the second part by the description of Cyproheptadine use (duration and frequency of use; reasons of use; sources of recommendation and procurement methods; mode of administration and the combination drugs used with Cyproheptadine). In the third part of the questionnaire, knowledge of the side effects of Cyproheptadine and users’ satisfaction were measured. The use of Cyproheptadine was measured by participants self-reported. In this study, the misuse of Cyproheptadine is definedas its use for gaining weight and having a roundness physical appearance and not for allergia, real anorexia, severe malnutrition or cachexia. Misusers were those participants, at the time of the study, who declared that they were currently using or who had ever used it within the past 6 months for that purpose.

### Statistical analysis

Statistical analyses were performed using the statistical package for social sciences (SPSS) of the University of Kinshasa version 20.0. Mean and standard deviation were used for quantitative variables and frequency and percentage for categorical variables. In order to determine the relationship between socio-demographic status and Cyproheptadine use Chi-square test was performed. Student *t*-test was used to compare means age of Cyproheptadine users and non-users. Logistic regression was used to determine predictors of Cyproheptadine use. A *p*-value of <0.05 was considered statistically significant.

## Results

### Characteristics of the study participants and frequency of Cyproheptadine use

A total of 499 participants were enrolled in the study, the response rate was 100 %. There were 352 females (70.6 %) and 147 males (29.4 %). Participants had a mean age of 24.9 years and a standard deviation of 9.7 years. As shown in Table 1, the majority of the study participants (72.9 %) were currently using or had used within the past six months Cyproheptadine as an appetite stimulant. The misuse of Cyproheptadine was significantly associated, as shown in Table 1, with sex, females (88.9 %) used more Cyproheptadine than males (34.7 %) (chi-square = 154.5, df = 1); with age (participants aged 13–19 (77.8 %) used more Cyproheptadine than those aged 36–55 (43.5 %)) (chi-square = 33.2, df = 3); with educational level (participants with no educational level (94.9 %) used more than participants with higher level (65.4 %)) (chi-square = 12.9, df = 2) and current marital status (participants who were currently married (67.7 %) used less than these who were never married (79.3 %) (Chi-square = 7.9, df = 2). The results of multivariate analysis are shown in Table 2. All statistically significant variables identified in the univariate analysis were included in the model which found two variables independently associated to misuse of Cyproheptadine, sex and age-groups. The model shows a statistically significant independent association between sex and the misuse of Cyproheptadine (wald chi-square = 87.7, df = 1), females were eleven times more likely to use Cyproheptadine (OR = 11.9; 95 % CI: 7.1–20.1) than males. This model also shows that the independent association between age-groups and the misuse of Cyproheptadine (wald chi-square = 7.2, df = 3). For the, people aged between 36 and 55 were three times less likely to use Cyproheptadine (OR = 0.3; 95 % CI: 0.2–0.8) compared to teenagers.

**Table 1:** Cyproheptadine use and sample characteristics, n (%)

Variables	Cyproheptadine use *n* = 499		*p*
	Users (*n* = 364)	Non users (*n* = 135)	
Sex			<0.001^a^
Male	51 (34.7)	96 (65.3)	
Female	313 (88.9)	39 (11.1)	
Total	364 (72.9)	135 (29.1)	
Age (years)			<0.001^a^
13–19	147 (77.8)	42 (22.2)	
20–24	70 (82.4)	15 (17.6)	
25–35	120 (73.6)	43 (26.4)	
36–55	27 (43.5)	35 (56.5)	
Highest educational level			0.008^a^
No education, preschool	37 (94.9)	2 (5.1)	
Primary	118 (75.6)	38 (24.4)	
Secondary	192 (69.1)	86 (30.9)	
Higher	17 (65.4)	9 (34.6)	
Current marital status			0.018^a^
Never married	165 (79.3)	43 (20.7)	
Currently married	176 (67.7)	84 (32.3)	
Formerly, ever married	23 (74.2)	8 (25.8)	
Age (mean ± SD)	Users (*n* = 364)	Non users (*n* = 135)	p
Male	23.88 ± 7,91	29.64 ± 12.42	0.003^b^
Female	23.70 ± 8.98	23.74 ± 6.37	0.975^b^

^a^Chi^2^ test

^b^ *t* test

**Table 2:** Socio-demographic determinants of the misuse of Cyproheptadine in Kinshasa

Variables	*P*	OR	95 % C.I.	
			Lower	Upper
Sex	<0.001^a^			
Male		1		
Female		11.9	7.1	20.1
Age groups	0.059^a^			
13–19		1		
20–24	0.556^a^	0.9	0.3	1.8
25–34	0.324^a^	0.7	0.4	1.4
35–55	0.007^a^	0.3	0.2	0.8

^a^Wald Chi^2^ test

### Duration and frequency of Cyproheptadine use

Table 3 shows that around half of the users declared to take Cyproheptadine for more than 12 months (50.5 %) and 69.0 % take it that daily.

**Table 3:** Characteristics of the use of Cyproheptadine

Sources of recommendation of the use of Cyproheptadine (*n* = 364)	
Friends	175 (48.1)
Media/self-reading	154 (42.3)
Relatives	84 (23.1)
Schoolmates	50 (13.7)
Health workers /pharmacists	47 (12.9)
Colleagues	17 (4.7)
Others sources	41 (11.2)
Reasons of use Cyproheptadine (*n* = 364)	
Anorexia	80 (22.0)
To gain weight	284 (78.0)
Mode of administration (*n* = 364)	
Oral	215 (59.1)
Anal	149 (40.9)
Frequency of use (*n* = 364)	
Daily	251 (69.0)
Weekly	45 (12.4)
Twice a week	7 (1.9)
Three times per week	8 (2.2)
Twice a month	14 (3.8)
Rarely	39 (10.7)
Duration of use (months) (*n* = 364)	
< 1	17 (4.7)
1–3	48 (13.2)
4–6	45 (12.4)
7–12	70 (19.2)
> 12	184 (50.5)
Procurement methods (*n* = 364)	
Medical prescription	27 (7.4)
Self-medication	337 (92.6)
Associate Cyproheptadine with others drugs	225 (61.8)
Drugs associated to Cyproheptadine (n =225)	
Dexamethazone	197 (87.6)
Vitamins	28 (12.4)

### Reasons and source of recommendation of the use of Cyproheptadine, procurement methods

Table 3 also shows that the main reason for Cyproheptadine use was weight gain (284/364 (78 %). The main recommended sources of Cyproheptadine mentioned by Cyproheptadine users were firstly, friends (48.1 %). Health workers/pharmacists were mentioned only by 12.9 % of users. Self-prescription was the most common procurement method (92.6 %), and only 7.4 % of users declared to have used a medical prescription for the procurement of Cyproheptadine.

### Mode of administration, and the combination drugs used with Cyproheptadine

The main mode of administration was oral (59.1 %), followed by the anal route (40.9 %). One hundred and thirty nine Cyproheptadine users (38.2 %) used Cyproheptadine on its own. Two hundred and twenty five users (61.8 %) reported to take Cyproheptadine associated with other drugs, Dexamethasone was the most common drug combined with Cyproheptadine (87.6 %).

### Knowledge of side effects of Cyproheptadine abuse, fear of addiction and satisfaction

As shown in Table 4, the majority of Cyproheptadine users (96.7 %) were aware that Cyproheptadine could give them some side effects. Overall, 77.2 % of Cyproheptadine users were afraid of the addictive effect of Cyproheptadine usage. However, the majority of users declared to be satisfied (97.5 %).

**Table 4:** Knowledge about side effects of misuse of Cyproheptadine and users’ satisfaction

Variables	n (%)
Knowledge of side effects of Cyproheptadine abuse (*n* = 364)	
Yes	352 (96.7)
No	12 (3.3)
Fear of addiction (*n* = 364)	
Yes	281 (77.2)
No	83 (22.8)
Satisfaction (*n* = 364)	
Yes	355 (97.5)
Not/not really	9 (2.5)

## Discussion

This study aimed to determine the prevalence of Cyproheptadine misuse in the Kinshasa population and to describe its characteristics. The main findings of this study were as follows: (i) the prevalence of Cyproheptadine misuse was 72.9 %. (ii) Cyproheptadine was used more by females than males and the Cyproheptadine use decreased with age. (iii) The majority of Cyproheptadine users reported to use Cyproheptadine on a daily basis. (iv) Half of Cyproheptadine users had used Cyproheptadine for a period over a year. (v) Self-prescription is by far the main procurement method of Cyproheptadine by the users. (vi) Dexamethasone was the most common drug combined with Cyproheptadine by users.

In the Literature, we did not find any paper that discussed Cyproheptadine use in the context of this study. In this study, then, the prevalence of Cyproheptadine misuse will be discussed with the prevalence of others drugs inappropriately used for increasing the body weight, muscle mass, strength in adults and an improved appearance. The most reported drug is androgenic-anabolic steroid which was mostly used by athletes and teenagers [21, 22]. The prevalence of Cyproheptadine misuse reported by this study was high comparatively to the prevalence of androgenic-anabolic steroid use reported in previous studies [22–24]. The prevalence difference may be explained by the price of Cyproheptadine which is cheaper than androgenic-anabolic steroid.

The medical prescription was less mentioned by users as the procurement method of Cyproheptadine, it corroborates with previous studies that also reported self-prescription as the most common procurement method of androgenic-anabolic steroid [21]. This study reported the long term misuse of Cyproheptadine, even though the information about the intake posology has been collected, it has not been reported because of the various pharmaceutical forms taken by users and the lack of precision in the posology. From the Literature, Cyproheptadine can reportedly produce serious side effects especially when taken in overdose; that includes hallucinations, convulsions, central nervous system depression and sudden cardiac arrest [25]. Death cases were also reported as a result of Cyproheptadine intoxication at a concentration of more than 15 times that of therapeutic. On routine daily use, Cyproheptadine may cause several side effects, including drowsiness, tired feeling, insomnia, spinning sensation, blurred vision, loss of coordination, upset stomach, nausea, diarrhea, and weight gain. Importantly, as previously described, many randomized controlled trials have demonstrated that Cyproheptadine can potentially induce weight gain and/or obesity [21–24]. We believe that the users of Cyproheptadine in Kinshasa are exposed to these risks].

It is known that a long-term corticotherapy can cause side effects such as hyperglycemia and diabetes, oedema, weight gain, hypertension and immune suppression [26]. In this study, the majority of Cyproheptadine users declared to take Cyproheptadine associated with Dexamethasone. As each of these two drugs independently induce weight gain and obesity, their combination can potentially increased weight gain and/ or obesity, thus, enhancing the occurrence of NCDs.

The knowledge of the side effects of Cyproheptadine does not prevent the use of Cyproheptadine because knowledge alone does not explain behaviour. Perception of risk is a major component of behaviour [27]. However human behaviour on issues such as body image is not always rational. Rationality depends on what a person considers to be of importance, this is also proven by this study which reported that even people with a high level of education taking Cyproheptadine. It is well established that females are more obese than males. A study conducted in South Africa showed that two factors can explain this difference in developing countries, childhood circumstances and adult socioeconomic status (SES) [28]. Evidence suggests that obesity independently increases the risk of CVD in women even in the absence of other metabolic abnormalities [29]. Despite a higher risk of obesity, females are more exposed to other obesogenic life style such as physical inactivity and sedentary behaviour [30]. This study also found that females were more frequent inappropriate users of Cyproheptadine than males. This high attraction to Cyproheptadine use by women can be explained by the fact that African women think that Black men find bigger women more attractive as described by Shoneye [16]. This hypothesis can be confirmed because our study showed that married women used Cyproheptadine less than single women probably because the single women will be more constrained to want to attract men.

This study reported that the misuse of Cyproheptadine decreases with age. Teenagers were among the age categories using more Cyproheptadine. Previous studies showed that the teenage years is a period during which teenagers, in order to fit their environment’s perception of beauty, are more concerned with their body weight, shape and size. Therefore, depending on the social context and social perception of beauty, teenagers may engage either in the weight loss or gain process. The perception of their body image is a key determinant of their dietary habits and weight management [31]. In the Congolese context, where plumpness is perceived as/considered a sign of beauty and wellbeing, young ladies usually engage in obesogenic behaviour. This may explain the use of Cyproheptadine by teenagers in our study.

In contrast with a study carried out in Accra, the capital of Ghana, less than ten percent of women reported to try to increase their body mass by eating more foods. This difference can be explained by the fact that the majority of women in the Ghana study (92. 2 %) reported that they were aware of the health risk associated with overweight and obesity and they were older than women in this study [32].

### Limitations and strengths of the study

This study has potential limitations. The two town sectors selected are not representative the whole population of Kinshasa; knowledge of the participants or their perception of risk for obesity and its complications were not assessed. Also, the effect of Cyproheptadine on weight gain and obesity was not measured during the study. We surmise that the best design to show this association should be a prospective study. These issues need to be addressed in future studies. Nevertheless, this is the only study, to the best of our knowledge, which has described that phenomenon among the Congolese population of Kinshasa. Additionally, the multi-stage sampling and the multi-centricity of our study sample have improved the generalisability of the result.

## Conclusion

This study shows that the Kinshasa population is significantly misusing Cyproheptadine and is highly exposed to its risk; including obesity. There is an urgent need to educate the community on the danger of substance abuse, such as Cyproheptadine and steroids. More profoundly, every effort should be made to change the view or concept of the community regarding obesity.
